# A Context-Aware Edge-Based VANET Communication Scheme for ITS †

**DOI:** 10.3390/s18072022

**Published:** 2018-06-24

**Authors:** Chang An, Celimuge Wu, Tsutomu Yoshinaga, Xianfu Chen, Yusheng Ji

**Affiliations:** 1 Inner Mongolia Normal University, Hohhot 010010, China; 2 Department of Computer and Network Engineering, The University of Electro-Communications, Tokyo 182-8585, Japan; yoshinaga@uec.ac.jp; 3 VTT Technical Research Centre of Finland, FI-90571 Oulu, Finland; Xianfu.Chen@vtt.fi; 4 Information Systems Architecture Research Division, National Institute of Informatics, Tokyo 101-8430, Japan; kei@nii.ac.jp

**Keywords:** vehicular networks, VANET, edge computing, unicast communications, broadcast communications, context-aware communications, intelligent transportation systems

## Abstract

We propose a context-aware edge-based packet forwarding scheme for vehicular networks. The proposed scheme employs a fuzzy logic-based edge node selection protocol to find the best edge nodes in a decentralized manner, which can achieve an efficient use of wireless resources by conducting packet forwarding through edges. A reinforcement learning algorithm is used to optimize the last two-hop communications in order to improve the adaptiveness of the communication routes. The proposed scheme selects different edge nodes for different types of communications with different context information such as connection-dependency (connection-dependent or connection-independent), communication type (unicast or broadcast), and packet payload size. We launch extensive simulations to evaluate the proposed scheme by comparing with existing broadcast protocols and unicast protocols for various network conditions and traffic patterns.

## 1. Introduction

Due to the rapid increase of the number of cars in China, people are facing increasing traffic congestion, car accidents, and CO${}_{2}$ pollution, as well as longer commuting time and increasing difficulty in finding parking. The intelligent transportation systems (ITS) become more and more important for supporting day-to-day commuter mobility management, tourism industry as well as large city event (e.g., events such as football matches, music events, or even bigger events like Olympic games) or even at unexpected events (e.g. flood, earthquake, etc.). All these applications require an efficient networking architecture to collect/disseminate information from/to vehicles, and other sensors/actuators. Vehicular ad hoc networks (VANETs) are capable of utilizing decentralized communication technologies to support efficient collection and dissemination of traffic related information. However, due to the limited wireless resources, high mobility of vehicles, and possible large node density, it is difficult to provide a satisfactory throughput with the conventional networking technologies.

Recently, mobile edge computing is introduced to conduct computation, caching task at the edge nodes that are closer than the cloud in order to reduce the response delay and improve the throughput. Some studies have proposed the use of vehicles as edge nodes [1,2,3,4]. However, the existing works do not sufficiently discuss the selection of edge nodes in a decentralized network environment. The joint operation of route selection and edge selection is not discussed as well.

There have been many studies discussing the routing problem in VANETs [5,6,7,8,9,10,11,12,13,14,15,16,17,18,19,20]. However, the unicast routing problem and broadcast problem have been discussed separately. Different applications could possibly require different levels of quality of services (QoS). Therefore, it is particularly important to consider context information, such as communication type (unicast or broadcast), end-to-end connection-dependency, and packet payload size, in the route selection. In the unicast communications, frame retransmissions can be conducted at the MAC layer while this is impossible for broadcast communications. As a result, the edge node selection criteria for unicast and broadcast communications are totally different. If an application only cares about the throughput, a delay tolerant approach could be a solution [21]. The packet payload size is another important context information that should be considered for the route selection. A larger packet will face a higher packet loss probability. The performance of a communication system can be improved by taking into account the context information for the routing decision. However, the design of an edge-based approach that is capable of handling different types of applications with different context information is still an under-explored research issue.

In this paper, we propose a context-aware edge-based general communication scheme for data exchange in vehicular networks. The proposed scheme uses a distributed approach to select edge nodes which are responsible to conduct packet forwarding. As compared with the conventional approach, the edge-based approach could efficiently reduce the number of concurrent sender nodes by aggregating user traffics at the edge nodes (Here the number of concurrent sender nodes denotes the number of nodes that content for the same wireless resources). When different devices acquire the same content, content cashing can be easily achieved at edge nodes. Since edge nodes are selected by considering the context information of the communications, the proposed scheme is able to handle various traffic patterns including unicast communications and broadcast communications. This paper is an extension of our previous conference paper [22]. This paper improves [22] by introducing a multi-hop data virtualization approach for connection-independent communications, and also presents new simulation results to show the performance advantage of the proposed approach over existing studies in a more comprehensive manner. The main contributions of this paper are as follows.

- We propose a context-aware general edge-based scheme that is possible to handle different types of communications with different requirements.
- The proposed scheme employs a fuzzy logic-based edge node selection protocol to find the best edge nodes in a decentralized network, which can achieve an efficient use of wireless resources by conducting packet forwarding through edges.
- The proposed scheme also uses a reinforcement learning algorithm to learn the best route for the last two-hop communications.
- We launch extensive simulations to evaluate the proposed scheme by comparing with other baselines.

## 2. Related Work

The existing studies cover broadcast protocols, unicast routing protocols, and vehicular edge computing approaches. Different types of communications with different context information possibly require different edges. An unified solution is required to handle different types of communications efficiently. However, there is no research addressing the design of context-aware route selection with edge assistance.

### 2.1. Vehicular Edge Computing

He et al. [1] have formulated the resource allocation strategy in vehicular networks as a joint optimization problem where the networking, caching and computing are taken into consideration. However, how to achieve the optimization in a decentralized network is not seriously addressed. Yuan et al. [2] have demonstrated that content caching at base stations and autonomous vehicles could improve the content delivery performance. Jutila [3] has proposed an admission control approach and a flow scheduling mechanism that work on network edges to optimize and control traffic flows and network resources. Wang et al. [4] have proposed a collaborative vehicular edge computing framework where a software defined network-based approach is employed to program, manipulate and configure network in a logically centralized way. While existing studies mainly focus on the use of caching and computation technologies to improve the networking performance, the joint route selection and edge selection problem in a decentralized network is not discussed adequately.

### 2.2. VANET Multi-Hop Broadcast Communications

A pioneer work on multi-hop broadcast protocols can be found in [5] where three different receiver-oriented approaches are proposed. Lai et al. [6] have proposed an average packet loss rate analysis model for vehicle-to-vehicle multi-hop broadcasting taking into account the mobility of vehicles, wireless channel conditions, and media access control (MAC). Rodriguez et al. [7] have proposed a streamwise queuing system on the receiver side to filter out the unimportant messages in a congestion situation. Alotaibi et al. [8] have proposed area-based dissemination protocols in vehicular networks with heterogeneous transmission powers. In the area-based dissemination protocols, a node that has higher potential new coverage area transmits first. Bi et al. [8] have proposed a protocol that takes into account the road layout in the forwarding decision, where three different types of broadcast decisions, namely the directional broadcast, bi-directional broadcast and multi-directional broadcast, are defined. A network coding-based approach to reduce the broadcast overhead has been proposed in [10]. Nguyen et al. [11] have proposed a hybrid TDMA/CSMA protocol that adjusts the length of broadcast frames based on three-hop neighbor information.

### 2.3. VANET Multi-Hop Unicast Communications

Many protocols have been proposed to take into account the vehicle mobility, route length, and inter-vehicle wireless link quality for the route selection since the performance of a communication route is affected by these multiple factors. Goudarzi et al. [14] have proposed a traffic-aware position-based routing protocol for city environments. The protocol uses small control packets to sample traffic conditions and makes routing decisions based on congestion conditions. In [15], the regularity of vehicle moving behaviors has been used to improve the routing performance by predicting a vehicle’s future locations based on the past traces and a hidden Markov model. Darwish et al. [16] have discussed the routing decision at the intersection, and proposed a protocol that makes routing decision based on road structure, vehicle position and received signal strength. There have been several studies considering some special types of vehicles to improve the routing. Sun et al. [18] have proposed a bus-based street-centric routing approach that uses buses as the main relay nodes for the packet forwarding. Similarly, [19] uses the bus systems as routing backbones of VANETs. In [20], drones are used for boosting VANET communications.

However, these existing approaches do not adequately address the MAC layer contention issue in a high-density network. Since vehicles could be deployed in a high-density manner for some hours or some road segments, the number of concurrent sender nodes is expected to be large. IEEE 802.11p, the standard for wireless access in vehicular environments, has the performance degradation problem when the number of sender nodes increases due to the MAC layer contention scheme based on the exponential backoff [12,13]. However, this problem is not sufficiently considered in the design of routing protocols.

## 3. Propose Scheme

### 3.1. Assumptions and Design Principles

Each vehicle (node) is able to get its own position information and road map through positioning services. Hello messages are exchanged periodically (1 Hz by default) to share location information and velocity information between neighbors.

The proposed scheme uses edge nodes to forward data packets. To efficiently utilize wireless resources, the scheme uses the same edge nodes for different traffic flows as far as possible. However, as different applications could expect different levels of QoS, the scheme also employs a context-aware approach to differentiate the traffic flows with different requirements. The proposed scheme generates different virtual edge nodes for different traffic flows with different context information. While the conventional routing protocols use the same route for the traffic flows with the same source-destination pairs, the proposed scheme could possibly use different routes as the edge nodes depending on the context information. We explain the proposed scheme by using three different types of communication patterns, namely end-to-end connection-dependent unicast, end-to-end connection-independent broadcast, and end-to-end connection-independent unicast communications.

### 3.2. Edge Node Selection and Packet Forwarding with Edge Nodes

**Figure 1:**
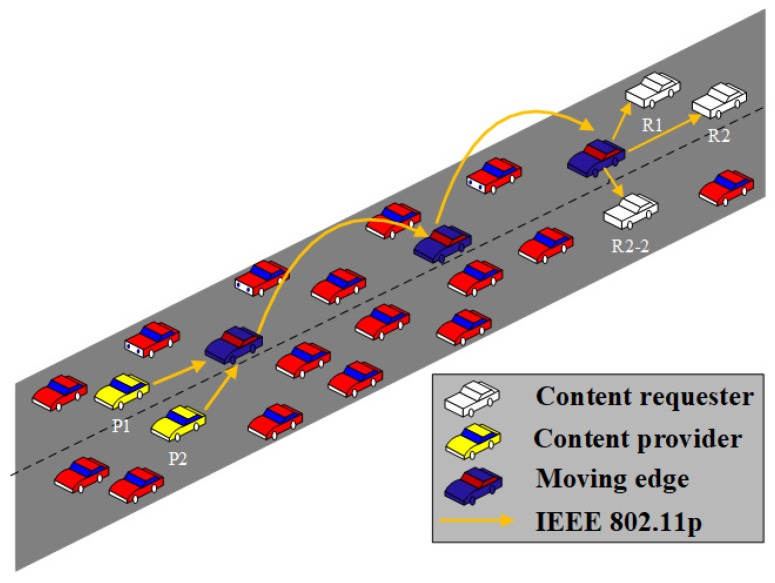
Edge-based forwarding.

Here, vehicles are used as edge nodes which conduct packet forwarding, computing, and content caching. We propose a concept that uses the same forwarder nodes for different traffic flows. As shown in Figure 1, three different traffic flows, specifically “P1→R1”, “P2→R2”, and “P2→R2-2”, use the same edges nodes for packet forwarding. The use of the same forwarder nodes results in two main benefits. First, the efficiency of wireless resource utilization is improved because the number of sender nodes can be reduced. Second, content caching at edge nodes becomes easier, resulting in a higher performance.

### 3.3. Criteria for Edge Node Selection

The proposed scheme selects the edge nodes using a distributed approach. The vehicle velocity, the vehicle distribution, and the channel conditions (link qualities) between the edge nodes and ordinary nodes are taken into account for the edge node selection. The consideration of vehicle velocity is to select slower vehicles as possible in order to avoid the overhead that could possibly occur due to the frequent change of edge nodes. The vehicle distribution is considered using the number of neighbors moving to the same direction which can reflect the long-term vehicle movement (in a two-way road, the edge nodes should be selected from the vehicles that have more vehicles moving toward the same direction). The link quality is also an important metric because a node that has better connections (for example, higher antenna height) with other non-edge nodes is preferred. A fuzzy logic-based approach is used to integrate these three metrics in the evaluation of the fitness for being an edge node.

In the proposed scheme, the vehicle velocity and the number of neighbor vehicles driving to the same direction are exchanged by hello messages. Each node calculates a competency value (as being an edge) for itself and each neighbor vehicle. If the node has the largest competency value in its vicinity ($\frac{R}{2}$ where *R* is the average transmission range), the node claims itself as an edge node in the next hello message. Edge node selection is conducted on a road segment basis which is defined in [23]. This ensures the connectivity between the selected edge nodes.

### 3.4. Calculation of Competency Value Based on Fuzzy Logic

A node becomes an edge node when it has the largest competency value in the neighborhood. A fuzzy logic-based algorithm is used to evaluate the competency value.

#### 3.4.1. Procedure

For each one-hop neighbor node, a node calculates a competency value as follows.

- **Calculation of the three factors:** Calculate the vehicle velocity, the number of vehicles moving to the same direction, and the channel condition information mentioned before.
- **Fuzzification:** Use predefined linguistic variables and membership functions to convert the factors to the corresponding fuzzy values.
- **Mapping and IF/THEN rule-based evaluation:** Map the fuzzy values to the predefined IF/THEN rules and combine the rules to get the rank of the neighbor as a fuzzy value.
- **Defuzzification:** Use a predefined output membership function and defuzzification method to convert the fuzzy value to a numerical value.

#### 3.4.2. Calculation of Multiple Factors

Upon reception of a hello message from a neighbor *x*, node *s* calculates the following factors.

**Velocity Factor (VF):** Node *s* extracts the velocity of node *x*, υ(x), and calculates VF(s,x) (the velocity factor for node *x* calculated at node *s*) as

(1) $$\begin{array}{c} VF\left(s,x\right)=\frac{\left|\upsilon \left(x\right)\right|-\min_{y\in N_{s}}\left|\upsilon \left(y\right)\right|}{\max_{y\in N_{s}}\left|\upsilon \left(y\right)\right|} \end{array}$$

where $N_{s}$ is the set of neighbors for node *s*. A lower VF indicates a lower velocity. VF is updated for every hello interval using a weighted exponential moving average as

(2) $$VF_{i}\left(s,x\right)\;\leftarrow \;\left(1-\alpha \right)\times VF_{i-1}\left(s,x\right)+\alpha \times VF_{i}\left(s,x\right),$$

where $VF_{i-1}\left(s,x\right)$ and $VF_{i}\left(s,x\right)$ denote the previous VF value and current VF value respectively. VF is initialized to 1. The coefficient α is set to 0.7 which is the best value for most situations according to our simulation results [the same value is used in Equations (4) and (5)].

**Follower Density Factor (FDF):** Node *x* announces the number of neighbor vehicles (c(x)) driving to the same direction by using hello messages. FDF of node *x* is calculated as

(3) $$\begin{array}{c} FDF\left(s,x\right)=\frac{c(x)}{\max_{y\in N_{s}}c\left(y\right)}. \end{array}$$

FDF indicates the vehicle density moving towards the same direction. A higher FDF is preferable as it means that the node is more suitable for being an edge node. FDF is initialized to 0, and updated for each hello interval using a weighted exponential moving average as

(4) $$FDF_{i}\left(s,x\right)\;\leftarrow \;\left(1-\alpha \right)\times FDF_{i-1}\left(s,x\right)+\alpha \times FDF_{i}\left(s,x\right).$$

**Channel Condition Factor (CCF):** For simplicity, the hello packet reception ratio is used as the channel Condition Factor (CCF). We only calculate the message reception ratio of the hello messages sent by the nodes located in *R* where *R* is the average transmission range. The hello messages are sent periodically with a predefined interval (1 s by default). If a vehicle has a better channel condition than other vehicles (for example, a truck with higher antenna), the CCF is larger. The CCF is initialized to 0, and updated as

(5) $$CCF_{i}\left(s\right)\leftarrow \left(1-\alpha \right)CCF_{i-1}\left(s\right)+\alpha \times CCF_{i}\left(s\right).$$

#### 3.4.3. Fuzzification

**Figure 2:**
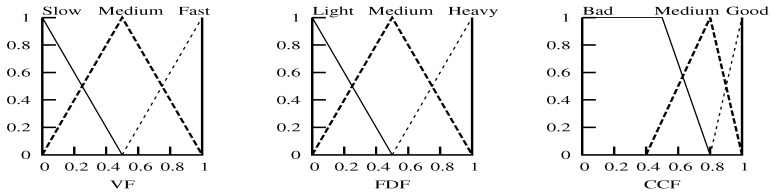
Fuzzy membership functions ((**left**): VF, (**middle**): FDF, (**right**): CCF).

Figure 2 shows the fuzzy membership functions for the velocity factor, follower density factor and channel condition factor. The velocity membership function defines what degree the velocity factor belongs to {Slow, Medium, Fast}. Similarly, the follower density membership function defines what degree belongs to {Heavy, Medium, Light} and what degree the channel condition factor belongs to {Good, Medium, Bad}.

#### 3.4.4. Mapping and Combination of IF/THEN Rules

Each node calculates the rank of the vehicle based on the IF/THEN rules (see Table 1). Since multiple rules could apply at the same time, the Min-Max method is used to combine their evaluation results (the same as the method used in [23]).

#### 3.4.5. Defuzzification

**Figure 3:**
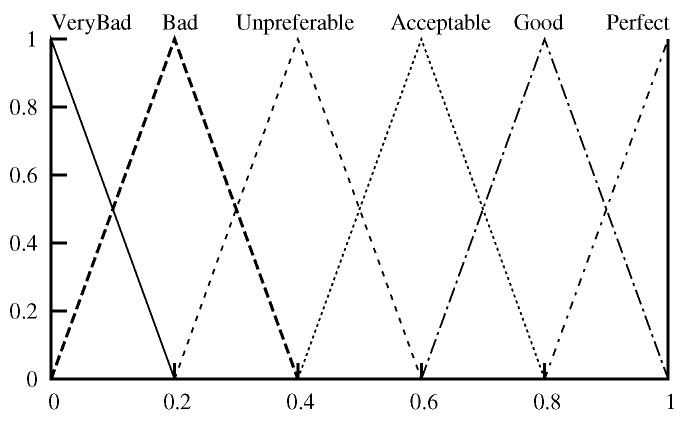
Output membership function.

The output membership function used is as shown in Figure 3. The Center of Gravity (COG) method is used in the defuzzification.

### 3.5. Last Two-Hop Route Optimization with Reinforcement Learning

**Figure 4:**
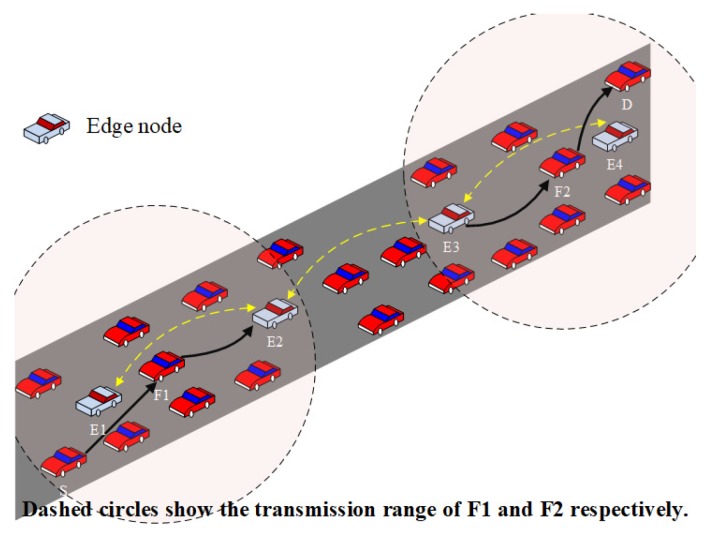
Last two-hop optimization.

The proposed scheme uses edge nodes to forward packets. As a result, different traffic flows could use the same edge nodes to forward packets assuming that the context information is the same (packet size and transmission type). This is efficient for utilizing wireless resources by reducing the number of sender nodes. However, this could increase the number of hops if the source (destination) node is very close from the next/previous edge node. To make the routing more efficient, we utilize a reinforcement learning-based algorithm to optimize the last two-hop route from/to the source/destination node. As shown in Figure 4, since E1, E2, E3, and E4 are the edge nodes, the default route from the source node (S) to the destination node (D) is “S→E1→E2→E3→E4→D”. By using the last two-hop optimization, the route can be optimized to “S→F1→E2→E3→F2→D” which is more efficient than the default route.

#### 3.5.1. *Q*-Learning Model

We define the *Q*-Learning algorithm as follows (Table 2). The entire network is the environment. Each packet P(o,r), indexed by its originator node *o* and the reference node *r* (the destination node or an edge node) is an agent. A node selects the next hop that it should forward a packet to. Hence the possible set of actions allowed at the node is the set of one-hop neighbors. Every node maintains a *Q*-Table which consists of *Q*-value [Q(r,x)] whose value ranges from 0 to 1, where *x* is the next hop to the reference node.

#### 3.5.2. Update of *Q*-Values

For each one-hop neighbor, a node maintains a value, ls(c,x), which shows the link status between node *c* and *x*. For simplicity, we use hello reception ratio to estimate the link status. However, the estimation can be improved by taking into account the vehicle velocity in the link status evaluation. The *Q*-Table is updated upon the reception of hello messages. Each node needs to maintain a *Q*-value for each one-hop neighbor, the destination (the traffic source node for the TCP ACK messages) node, and the edge nodes located in two-hop distance. *Q*-values are broadcasted by each node using hello messages. Each *Q*-value is initialized to 0. Upon reception of a hello message from node *x*, node *c* updates the corresponding *Q*-value to the node *r* as

(6) $$\begin{array}{ccc} Q_{c}\left(r,x\right) & \leftarrow & \hat{\alpha }\times ls\left(c,x\right)\times \left\{\hat{R}+\gamma \times \max_{y\in N_{x}}Q_{x}\left(r,y\right)\right\}\\ & & +\;\left(1-\hat{\alpha }\right)\times Q_{c}\left(r,x\right). \end{array}$$

**Figure 5:**
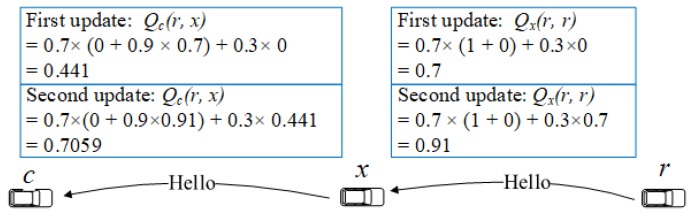
An example for *Q*-value update.

The learning rate ($\hat{\alpha }$) is 0.7, and the discount factor (γ) is 0.9. $\max_{y\in N_{x}}Q_{x}\left(r,y\right)$ is the maximal *Q*-value of *x* to node *r*. The reward $\hat{R}$ is calculated as

(7) $$\hat{R}=\left\{\begin{array}{cc} 1, & \mathrm{if}\;c\in N_{r}\\ 0, & \mathrm{otherwise} \end{array}\right.$$

where $N_{r}$ denotes the one-hop neighbor set of node *r*. When node *c* is a neighbor of node *r*, the reward is 1 and otherwise 0. Please note that only one *Q*-value is maintained for each pair of state and action. Upon reception of a hello message, the corresponding *Q*-value is updated according to Equation (6) which discounts the reward with the increase of number of hops. This ensure that the hop count is considered in the route selection. The quality of each link is also taken into account by discounting the reward with the link status value [ls(c,x)]. As a result, a *Q*-value represents the evaluation of a next packet forwarder candidate with the consideration of multi-hop performance. Therefore, the proposed scheme is able to optimize the route to the reference node. Figure 5 shows an example for *Q*-value update where ls(·,·) is 1.

### 3.6. Context Acquisition and Utilization

**Figure 6:**
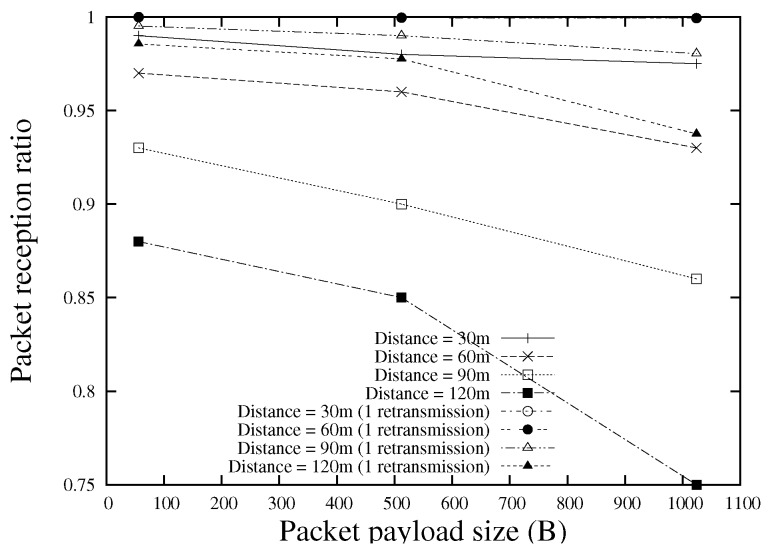
MAC broadcast frame reception ratio for various packet payload sizes.

Three different types of context information, namely connection-dependency (connection-dependent or connection-independent), communication type (unicast or broadcast), and packet payload size, are considered in the proposed scheme. More context information can be included with a simple extension. Figure 6 shows the MAC broadcast frame reception ratio for various packet payload sizes, where the data are achieved by conducting a real-world VANET experiment with IEEE 802.11 b/g/n devices. It is easy to observe the significant effect of packet size on the packet reception probability.

VANET applications can be classified into three different categories, specifically end-to-end connection-dependent unicast, end-to-end connection-independent broadcast, and end-to-end connection-independent unicast applications. Different applications have different requirements. Specifically, for broadcast applications, since there is no retransmissions at the MAC layer, a more reliable link between two communication nodes is required. For unicast applications, the requirement on the link quality could be looser than broadcast as frame retransmissions are possible to improve the reliability.

**Figure 7:**
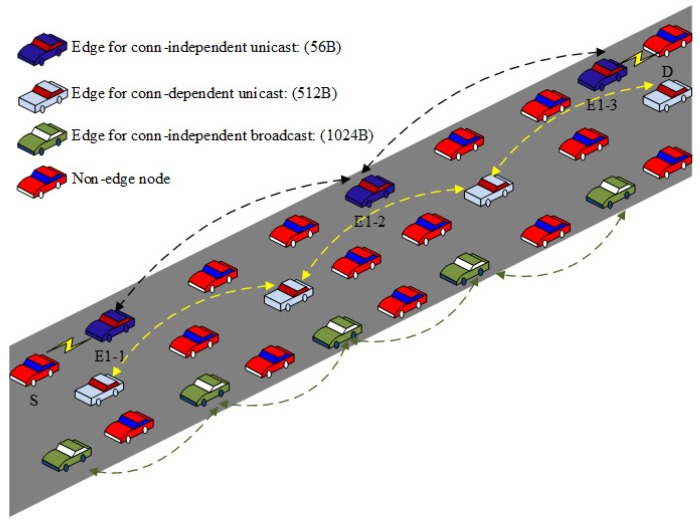
Context-aware edge selection.

In the proposed scheme, three different sizes of hello (probe) packets are used to estimate the frame reception probability for each size. The hello message size is randomly selected from {56, 512, 1024} (bytes) (this can be easily extended to support various payload sizes). This selection lasts for 100 s (This is a system parameter which could be tuned according to the network environment. If the node density is not sufficiently high, this value should be set to a smaller one, which is better to calculate the reception ratios for different sizes of hello packets in a short period. Please note that the value we use here (100 s) is acceptable for most scenarios according to our simulations, and the dynamic tuning of this parameter is beyond the scope of this paper). If the selected payload size is not sufficient to transmit all the data required, the node uses multiple packets to transmit. If the selected size is larger than the size required, the node uses zero padding. Link quality information is maintained for each possible hello payload size ({56, 512, 1024}) (bytes). Upon reception of a hello message, the link quality information is updated for the corresponding payload size. For unicast applications, the hello reception ratio (CCF in Equation (5)) is calculated by considering three retransmissions at the MAC layer. By using different sizes of hello packets, we can utilize the context information to improve the efficiency of packet forwarding process (see Figure 7).

### 3.7. Virtual Multi-Hop Data Delivery for Connection-Independent Unicast Applications

Most unicast applications in vehicular networks, such as software upgrading, car sensor data collection and so on, require a reliable end-to-end data delivery. TCP is a widely used end-to-end acknowledgment-based data delivery approach. In TCP, the sender node adapts the sending rate, namely congestion window size, by using a congestion avoidance algorithm according to the acknowledgment packets successfully received. The throughput of a TCP connection is affected significantly by the end-to-end packet loss probability. As a result, TCP has a performance degradation problem in a lossy multi-hop networks where a high end-to-end packet loss ratio restricts the increase of the congestion window size at the sender node.

**Figure 8:**
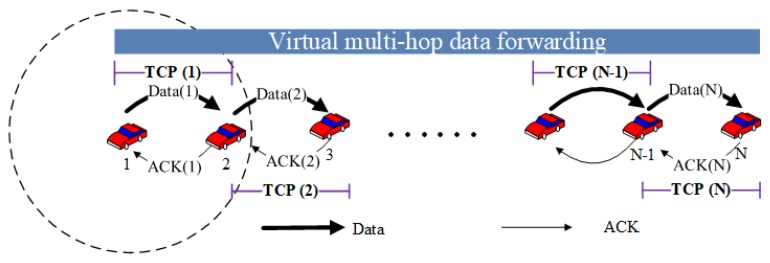
Virtual multi-hop data forwarding (dashed circle shows the transmission range).

The main purpose of sensor data collection applications is to send the data to the cloud. Therefore, in most cases, a real-time end-to-end feedback is not required. To solve the performance degradation problem of TCP in multi-hop networks, we use a multi-hop data delivery virtualization approach [24] to conduct multi-hop data transmissions through multiple one-hop TCP sessions. The virtualization approach works on the top of transport layer. As shown in Figure 8, a multi-hop data transmission from vehicle 1 to vehicle N is conducted by multiple one-hop data transmissions where each hop is managed by a TCP session, which ensures the reliability and fairness while facilitating an efficient use of wireless resources. Each forwarder node is responsible for transmitting data segments to the next hop which is closer to the destination. Since each one-hop communication is conducted based on TCP, the congestion window size at each TCP sender node is not affected by the number of hops between the source node and the destination node.

## 4. Simulation Results

Network simulator ns-2.34 [25] was used to conduct simulations in street scenarios (see Table 3). The channel fading was addressed by using Nakagami propagation model (see Table 4) [26]. The average transmission distance was approximately 250 m. The proposed scheme was compared with “Non-edge” (the conventional approach without edge computing), “Edge (BeaconSz = 56 B)” (Edge-based approach with beacon size of 56 bytes), “Edge (BeaconSz = 512 B)”, and “Edge (BeaconSz = 1024 B)”. The protocols were evaluated under broadcast traffic flows, connection-dependent unicast traffic flows, and connection-independent traffic flows. The error bars in the following figures indicate the 95% confidence intervals.

### 4.1. Connection-Dependent Broadcast Flows

**Figure 9:**
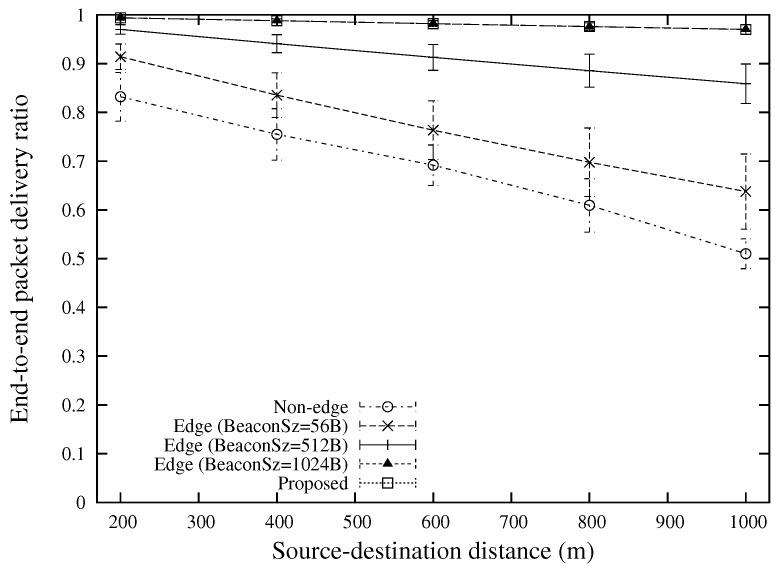
End-to-end packet delivery ratio for various source-destination distances (the case of 2 broadcast flows).

Figure 9 shows the end-to-end packet delivery ratio for various source-destination distances. All the edge-based approaches achieve significantly better performance than the conventional approach without edge computing. This is because the packet forwarding through the edge node could efficiently reduce the MAC layer contention by using smaller numbers of sender nodes, resulting in a better wireless resource utilization. When the beacon size is small (“Edge (BeaconSz = 56 B)”), it is difficult to achieve a high packet delivery ratio as the small size beacon overestimates the link quality. Since the proposed scheme uses different sizes of beacons for different requirements, the link quality is evaluated more accurately, and this contributes to a significant improvement of packet delivery ratio as compared with other approaches that use constant packet size for beacons.

**Figure 10:**
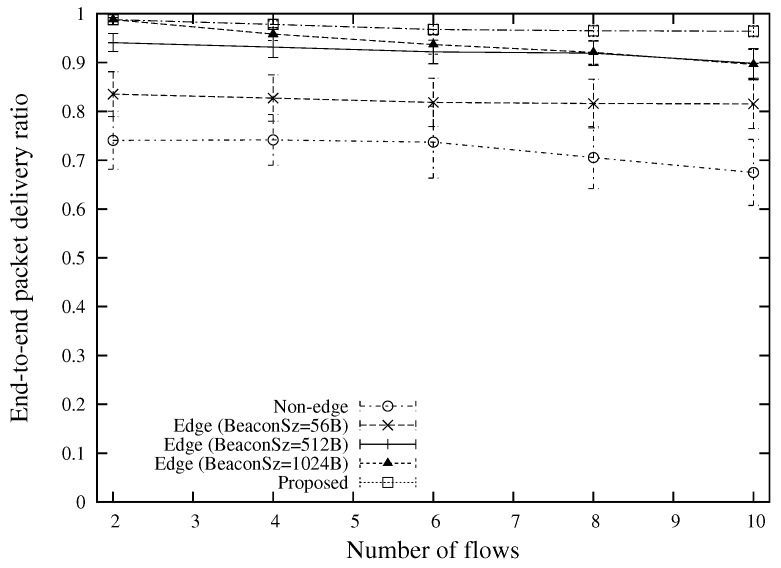
End-to-end packet delivery ratio for various numbers of flows (the case of 400 m source-destination distance).

The end-to-end packet delivery ratio for different numbers of concurrent unicast traffic flows is shown in Figure 10. Although “Edge (BeaconSz = 1024 B)” is able to provide a high packet delivery ratio, the performance drops drastically with the number of traffic flows as a large size beacon underestimates the link quality, resulting in a large number of hops than the optimal one. The proposed scheme is able to attain a better performance in various number of traffic flows by selecting routes with consideration of packet payload sizes. Another contributor for the high packet delivery ratio is the edge-based packet forwarding approach which could reduce the number of forwarder nodes as compared with the conventional non-edge approaches.

### 4.2. Connection-Dependent Unicast Flows

**Figure 11:**
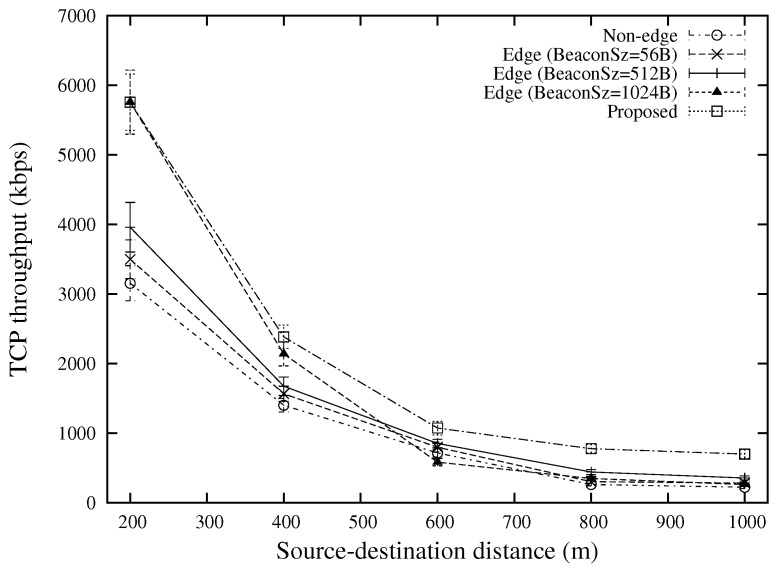
TCP throughput for various source-destination distances (the case of 20 TCP flows).

**Figure 12:**
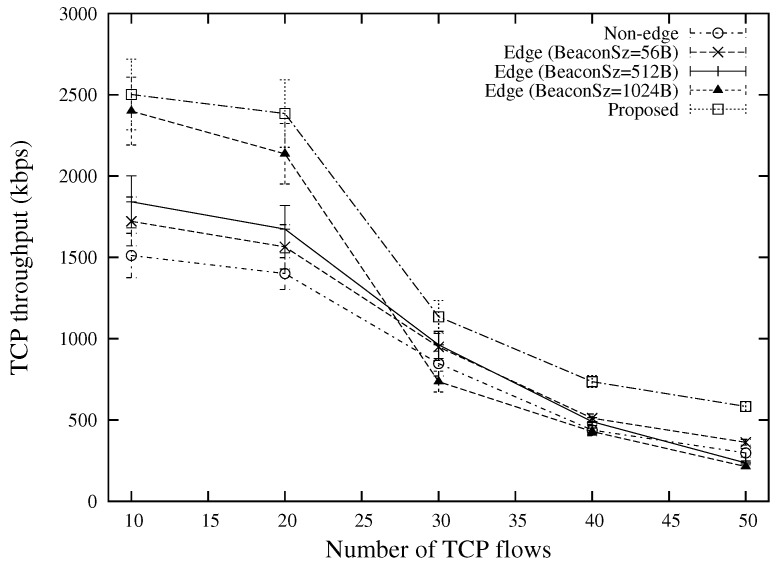
TCP throughput for various numbers of flows (the case of 400 m source-destination distance).

The TCP throughput under various source-destination distances and various numbers of flows are shown in Figure 11 and Figure 12, respectively. The best performance is attained by the proposed scheme. This is because by using different types of beacon messages, the proposed scheme can reduce the number of hops as compared with “Edge (BeaconSz = 1024 B)” and select better forwarder nodes that have better qualities as compared with “Edge (BeaconSz = 56 B)”. For an end-to-end TCP flow, the proposed scheme is able to select different routes for data packets and ACK packets, which can significantly shorten the round trip time as compared with the conventional approaches. The advantage over other approaches for a large number of traffic flows comes from the edge-based forwarding scheme which reduces the number of contender nodes for the same wireless resources.

### 4.3. Connection-Independent Unicast Flows

**Figure 13:**
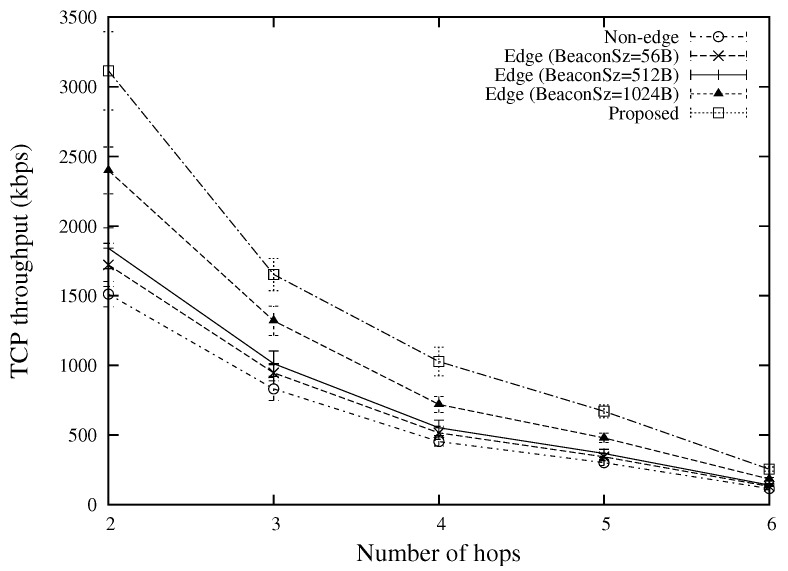
TCP throughput for various numbers of hops (the case of 10 TCP flows).

Figure 13 shows the TCP throughput for various numbers of hops. By using the virtual multi-hop data delivery, the proposed scheme can achieve more than 20 percent improvement over other approaches in all situations (“Edge (BeaconSz = 56 B)”, “Edge (BeaconSz = 512 B)”, and “Edge (BeaconSz = 1024 B)” do not use the virtual multi-hop data delivery). This is because the virtual multi-hop data delivery can result in a more efficient utilization of wireless resources by providing a higher congestion window size at each sender node as compared with the conventional end-to-end TCP approach.

## 5. Conclusions

We proposed a context-aware edge-based routing scheme that supports both the broadcast applications and unicast applications. The scheme uses a context-aware approach to generate edge nodes which are used to forward the data packets for the purpose of reducing the number of contender nodes for the same wireless resources. Some content information, including the connection-dependency, communication type, and packet payload size, are considered in the route selection. A reinforcement learning-based approach is employed to optimize the last two-hop routes in order to improve the efficiency of the end-to-end communication. The simulation results show that the proposed scheme can achieve a significantly better performance as compared with other baseline approaches for different types of communications with different packet sizes. 

## Figures and Tables

**Table 1:** Rule Base.

	Velocity	Follower Density	Channel Condition	Rank
Rule1	Slow	Heavy	Good	Perfect
Rule2	Slow	Heavy	Medium	Good
Rule3	Slow	Heavy	Bad	Unpreferable
Rule4	Slow	Medium	Good	Good
Rule5	Slow	Medium	Medium	Acceptable
Rule6	Slow	Medium	Bad	Bad
Rule7	Slow	Light	Good	Unpreferable
Rule8	Slow	Light	Medium	Bad
Rule9	Slow	Light	Bad	VeryBad
Rule10	Medium	Heavy	Good	Good
Rule11	Medium	Heavy	Medium	Acceptable
Rule12	Medium	Heavy	Bad	Bad
Rule13	Medium	Medium	Good	Acceptable
Rule14	Medium	Medium	Medium	Unpreferable
Rule15	Medium	Medium	Bad	Bad
Rule16	Medium	Light	Good	Bad
Rule17	Medium	Light	Medium	Bad
Rule18	Medium	Light	Bad	VeryBad
Rule19	Fast	Heavy	Good	Unpreferable
Rule20	Fast	Heavy	Medium	Bad
Rule21	Fast	Heavy	Bad	VeryBad
Rule22	Fast	Medium	Good	Bad
Rule23	Fast	Medium	Medium	Bad
Rule24	Fast	Medium	Bad	VeryBad
Rule25	Fast	Light	Good	Bad
Rule26	Fast	Light	Medium	VeryBad
Rule27	Fast	Light	Bad	VeryBad

**Table 2:** *Q*-learning model.

Environment	The Entire Vehicular Ad Hoc Network
Agent	Each packet P(o,r)
State of the agent	Each node in the network
State space	The set of all nodes in the network
Actions	Selecting an one-hop neighbor as the next hop

**Table 3:** Simulation Environment.

	Street Scenario
Topology	1700 m × 1700 m
Number of vehicles	619
Mobility generator	SUMO [27] + TraNS [28]
Packet size	randomly selected from {56 B, 512 B, 1024 B}
Broadcast data rate	15 packets per second
MAC	IEEE 802.11p (broadcast: 3 Mbps, unicast: 27 Mbps)
Propagation model	Nakagami Model
Simulation time	500 s

**Table 4:** Parameters of Nakagami Model.

gamma0_	gamma1_	gamma2_	d0_gamma_	d1_gamma_
2.0	2.0	2.0	200	500
m0_	m1_	m2_	d0_m_	d1_m_
1	1	1	80	200
